# Intradialytic aerobic cycling exercise alleviates inflammation and improves endothelial progenitor cell count and bone density in hemodialysis patients

**DOI:** 10.1097/MD.0000000000004134

**Published:** 2016-07-08

**Authors:** Min-Tser Liao, Wen-Chih Liu, Fu-Huang Lin, Ching-Feng Huang, Shao-Yuan Chen, Chuan-Chieh Liu, Shih-Hua Lin, Kuo-Cheng Lu, Chia-Chao Wu

**Affiliations:** a Department of Pediatrics, Taoyuan Armed Forces General Hospital, Taoyuan; b Department of Pediatrics, Tri-Service General Hospital, National Defense Medical Center, Taipei; c Department of Internal Medicine, Cardinal Tien Hospital, Yong He Branch, New Taipei; d School of Public Health, National Defense Medical Center, Taipei; e Department of Internal Medicine, Cardinal Tien Hospital, School of Medicine, Fu-Jen Catholic University, New Taipei; f Division of Nephrology, Department of Medicine, Tri-Service General Hospital, National Defense Medical Center, Taipei, Taiwan.

**Keywords:** bone density, endothelial progenitor cell, hemodialysis, inflammation, intradialytic exercise

## Abstract

Inflammation, endothelial dysfunction, and mineral bone disease are critical factors contributing to morbidity and mortality in hemodialysis (HD) patients. Physical exercise alleviates inflammation and increases bone density. Here, we investigated the effects of intradialytic aerobic cycling exercise on HD patients. Forty end-stage renal disease patients undergoing HD were randomly assigned to either an exercise or control group. The patients in the exercise group performed a cycling program consisting of a 5-minute warm-up, 20 minutes of cycling at the desired workload, and a 5-minute cool down during 3 HD sessions per week for 3 months. Biochemical markers, inflammatory cytokines, nutritional status, the serum endothelial progenitor cell (EPC) count, bone mineral density, and functional capacity were analyzed. After 3 months of exercise, the patients in the exercise group showed significant improvements in serum albumin levels, the body mass index, inflammatory cytokine levels, and the number of cells positive for CD133, CD34, and kinase insert domain-conjugating receptor. Compared with the exercise group, the patients in the control group showed a loss of bone density at the femoral neck and no increases in EPCs. The patients in the exercise group also had a significantly greater 6-minute walk distance after completing the exercise program. Furthermore, the number of EPCs significantly correlated with the 6-minute walk distance both before and after the 3-month program. Intradialytic aerobic cycling exercise programs can effectively alleviate inflammation and improve nutrition, bone mineral density, and exercise tolerance in HD patients.

## 1 Introduction

End-stage renal disease (ESRD) patients tend to have multiple comorbidities, especially cardiovascular disease, and mineral and bone disorder. Inflammation, endothelial dysfunction, and mineral bone disease (MBD) are critical factors contributing to morbidity and mortality in hemodialysis (HD) patients. Physical inactivity is a major factor contributing to chronic inflammation and protein-energy wasting. Consequently, physical inactivity and cardiovascular and bone morbidities can form a vicious cycle in ESRD patients.^[1]^ In theory, exercise prevents the side effects of inactivity and thus cardiovascular mortality. Recently, exercise has been shown to improve cardiovascular outcomes,^[2]^ dialysis efficacy,^[3]^ physical function,^[4]^ health-related quality of life,^[5]^ and high-sensitivity C-reactive protein (hs-CRP) levels^[6]^ in patients undergoing HD.

Circulating endothelial progenitor cells (EPCs) may facilitate regenerating the endothelial lining of damaged blood vessels, and, in 1 study, the number of EPCs correlated with the outcome in patients with heart attack.^[7,8]^ In HD patients, the number of EPCs was significantly lower than that in the general population.^[9]^ The mobilization and number of EPCs was also related to the capacity to exercise, suggesting a possible connection with the cardiovascular risk in low-performance populations limited by chronic diseases.^[10]^ Regular physical activity seems to increase the number and function of circulating EPCs, which might lead to improved cardiac function in patients with recently acquired acute myocardial infarction.^[11]^ In addition, exercise rehabilitation programs were suggested to improve the functional capacity of HD patients.^[12]^

End-stage renal disease patients have an extremely high risk of bone fracture because they have a high incidence of uremic osteoporosis and MBD. Compared with a normal population, uremic patients are more likely to have low bone mass, a disarranged microarchitecture, skeletal fragility, and abnormal bone metabolism.^[13]^ The mechanical loading applied to the bone by the muscle is directly responsible for bone formation and remodeling; hence, there is a positive correlation between muscle strength and bone mineral density (BMD). Exercise may promote bone growth and suppress bone loss through several mechanisms. Among the types of exercise training, aerobic training is considered more beneficial than resistance (or strength) training because of the additional benefit of reducing plasma hs-CRP levels further. Compared with interdialytic exercise, intradialytic exercise has more advantages including a lower participant dropout rate, higher motivation, and a safer, structured environment with close monitoring.^[12,14]^

In this study, we conducted an exercise rehabilitation program involving intradialytic aerobic cycling exercise to investigate whether physical exercise can improve inflammation, the EPC count, and BMD in HD patients.

## 2 Materials and methods

### 2.1 Study subjects

The study population was composed of 40 prospectively recruited, voluntary end-stage renal disease patients. Their ages ranged from 21 to 65 years. The Institutional Research Board of the Tri-Service General Hospital, Taipei, Taiwan, approved the study (TY101-15 and TY102-04). Written informed consent of each participant was obtained. They were all receiving maintenance HD for at least 6 months with 3 times a week and 4 hours for each session. All subjects completed a prestudy medical surveillance, including physical evaluations, electrocardiography, resting echocardiography, and blood biochemical tests. They were randomized to a 3-month intradialytic exercise program (group E: 20 patients) or a control group (group C: 20 patients). None of them had poor control hypertension, moderate to severe heart failure (New York Heart Association class ≥II), arrhythmia (>III according to Lown), recent heart attack or unstable angina, active liver dysfunction, or skeletal problems prohibit liberal exercise. Other exclusion criteria included the presence of active inflammation/infection, malignancy, autoimmune diseases, emotional instability, musculoskeletal disability, poorly controlled blood glucose or parathyroid hormone (PTH) levels, uncontrolled cardiac failure or respiratory problems, prior month hospitalization, use of medications that may influence inflammatory cytokine levels, the lower extremities arterial-venous access, and increased body weight with a body mass index more than 25 kg/m^2^.

The exclusion criteria were a presence of active infection or inflammation, autoimmune disorders, malignancy, psychiatric diseases, severe musculoskeletal disorders, poorly controlled diabetes or secondary hyperparathyroidism, uncontrolled heart failure or pulmonary diseases, hospitalization during the previous month, use of drugs that influence serum cytokine levels, vascular access in the lower extremities, and a body mass index higher than 25 kg/m^2^. All tests were conducted on a nondialysis day.

### 2.2 Intradialytic aerobic cycling exercise program

**Figure 1:**
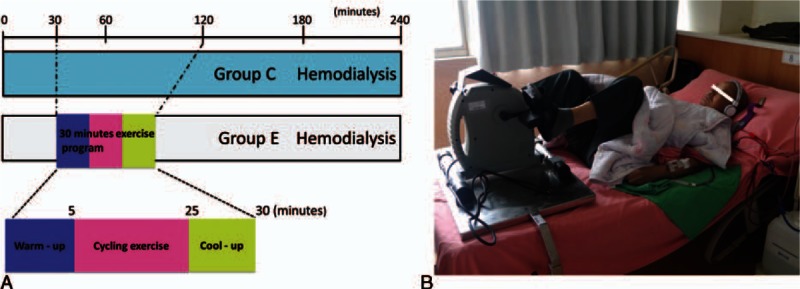
Study design flow chart and the intradialytic aerobic cycling exercise. A, Patients were randomized to either a 3-month intradialytic aerobic cycling exercise program completed during their hemodialysis sessions (group E: 20 patients) or the control status (group C: 20 patients). B, The intradialytic aerobic cycling devices were adjusted to each patient's bed for his or her own comfort and safety.

The patients in group E complied with a 30-minute exercise program that consisted of a 5-minute warm-up, 20 minutes of cycling at a desired workload, and a 5-minute cool down during their HD treatment in the renal unit (Fig. 1A). The initiative time was within 30 and 90 minutes, depending on the individual condition after basal stabilization. The duration of cycling may have gradually increased over time according to each patient's ability until reaching maximal duration. The frequency of exercise was 3 times weekly, 30 minutes each time in the first 2 hours of HD. The exercise was performed under the supervision of a physician and a nurse who specialized in rehabilitation. The intensity of exercise was approximately 12 to 15 on the Borg Perceived Exertion Scale. The patients’ cardiac rhythm was monitored continuously throughout the course of the HD session. Their blood pressure was also measured every 15 minutes. The cycling devices were adjusted to each patient's bed for his or her own comfort and safety (Fig. 1B).

### 2.3 Biochemistry analyses

Blood samples were drawn at the baseline and after the 3-month study. After 10 hours of fasting, blood was drawn at 8:00 to 9:00 AM and centrifuged within 30 minutes of collection. Fasting blood samples were obtained from the patients before the midweek dialysis session at the baseline and after the 3rd, 6th, and 12th month. After collection, serum samples were immediately frozen and stored at −70^o^C until use. Total calcium, serum phosphate, total alkaline phosphatase, and serum albumin were evaluated using an AU5000 automated chemistry analyzer (Olympus, Tokyo, Japan). Fasting blood samples were also analyzed for levels of glucose, total cholesterol, high-density lipoprotein (HDL)-cholesterol, and triglycerides. Serum levels of interleukin (IL)-6 were measured using the enzyme-linked immunosorbent assay method (R&D Systems, Minneapolis, MN).

### 2.4 Endothelial progenitor cell analysis

Endothelial progenitor cells were assessed within 1 hour of collection. Briefly, 10 mL of blood was drawn from the antecubital vein of the patients and collected in a 4-mL tube containing heparin. Mononuclear cells (MNCs) were then isolated through density-gradient centrifugation by using Ficoll 400 (Ficoll-Paque PLUS, Amersham Biosciences, Sweden). The MNCs were washed twice with phosphate buffer solution (PBS) and centrifuged before resuspension with 300 μL of PBS. A cell viability of >95.0% was required in each group. EPCs were identified by the coexpression of different stem cell markers (CD34, CD133) and the endothelial cell lineage marker kinase insert domain-conjugating receptor (KDR). The EPCs in peripheral blood were identified through flow cytometry by using triple staining. To identify the expression of the EPC surface markers CD133, CD34, and KDR, peripheral blood MNCs (4 × 10^5^) were incubated for 30 minutes with monoclonal antibodies against allophycocyanin (APC)-conjugated CD133, fluorescein isothiocyanate 19 (FITC)-conjugated CD34 and phycoerythrin (PE)-conjugated KDR at room temperature in a dark room. Quantitative 3-color flow cytometric analysis was performed using a fluorescence-activated cell sorter (Cytomic FC 500 system; Beckman Coulter Inc.).

### 2.5 Bone mineral density

Serum intact PTH (iPTH) levels were measured through radioimmunoassay. BMD was measured using dual-energy X-ray absorptiometry (DEXA) at the lumbar spine (L2–L4) and right proximal femur (femoral neck and trochanter). The DEXA was performed at the onset of the study and again 1 year after initiation of the study. All scans and analyses were performed by the same operator with a coefficient of variation of 1.5% at both the lumbar spine and femoral neck.

### 2.6 The 6-minute walk test

The 6-minute walk test is a safe, easy to administer, and tolerable test, and can accurately mirror the activities of daily living. Thus, we use the 6-minute walk distance (6-MWD) as the primary measure. When conducting the test, we did not assist the patient in carrying or pulling supplemental oxygen. The patient should walk by himself/herself. We did not use a treadmill for testing. We also did not use an oval or circular track.^[15]^ We did not use an oval or circular track. The 6-MWD is a useful measure of functional capacity, targeted at people with at least moderately severe impairment. It has been widely used to measure the response to therapeutic interventions for clinical scenarios.

### 2.7 Statistical analysis

Continuous data were expressed as the mean ± standard deviation and were analyzed using the paired *t* test. Categorical data were expressed as frequencies (%) and were evaluated using the chi-square test or Wilcoxon test. Data including systolic blood pressure, diastolic blood pressure, and heart rate were evaluated using the repeated-measures analysis of variance (ANOVA) test. One-way ANOVA was used to assess the significance of various characteristics, laboratory data, and adverse outcomes. The data were analyzed using the Statistical Package for the Social Sciences version 17.0 statistical software (SPSS, Inc., Chicago, IL), and differences with *P* values <0.05 were considered statistically significant.

## 3 Results

We enrolled 40 maintenance HD patients (group E, n = 20; group C, n = 20) in the study which comprised 23 females and 17 males. The mean age (±SD) was 62 ± 8 years. There were no significant differences in the demographic characteristics of the study subjects (Table 1). No obvious symptoms or tiredness was reported during any of the HD sessions.

**Table 1:**
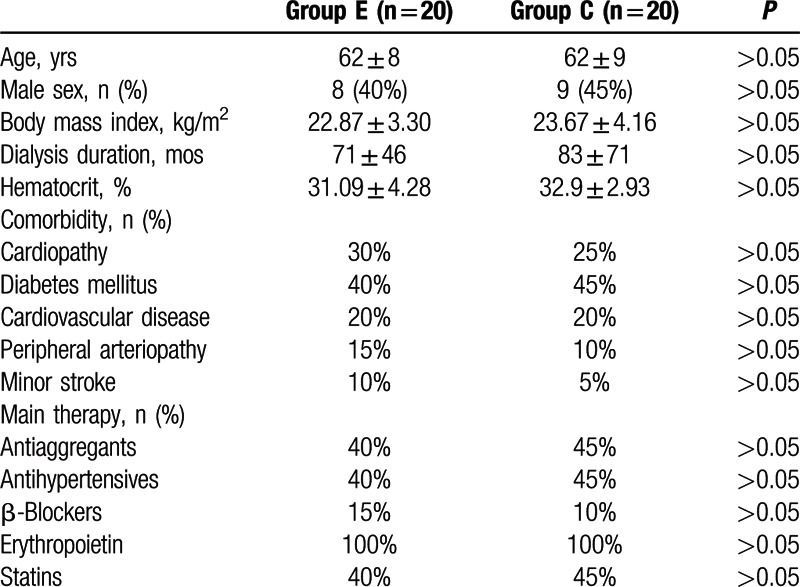
Baseline characteristics of hemodialysis patients who were prescribed a 3-month exercise program (group E) or not prescribed exercise (group C) and completed the study.

There were no differences among comorbidities including diabetes mellitus, hypertension, stroke, and peripheral arteriography between groups C and E. There were also no differences in the major medical treatments, including antiaggregants, antihypertensive drugs, β-blockers, statins, and erythropoietin between groups C and E.

### 3.1 The changes of blood pressure and heart rate during the intradialytic aerobic cycling exercise

There were no differences of baseline systolic blood pressure, diastolic pressure, and heart rate between groups C and E. For acute physiologic response to exercise, there were significantly increased systolic and diastolic blood pressure and heart rate during intradialytic aerobic cycling exercise in group E compared with those before exercise. All these changes were within physiologic range and no obvious discomforts were complained. Apart from this, for chronic physiologic response to exercise, after the 2 to 3 months training of intradialytic aerobic cycling exercise, obviously attenuated systolic and diastolic blood pressure and heart rate during pre-exercise basal period were also revealed. Comparing group E with group C, the significantly increased systolic blood pressure and heart rate during intradialytic aerobic cycling exercise were found within the initial 2 months. After the intradialytic aerobic cycling exercise for 3 months, the effect was attenuated and no obvious difference of blood pressure and heart rate was found during the third month (Table 2).

**Table 2:**
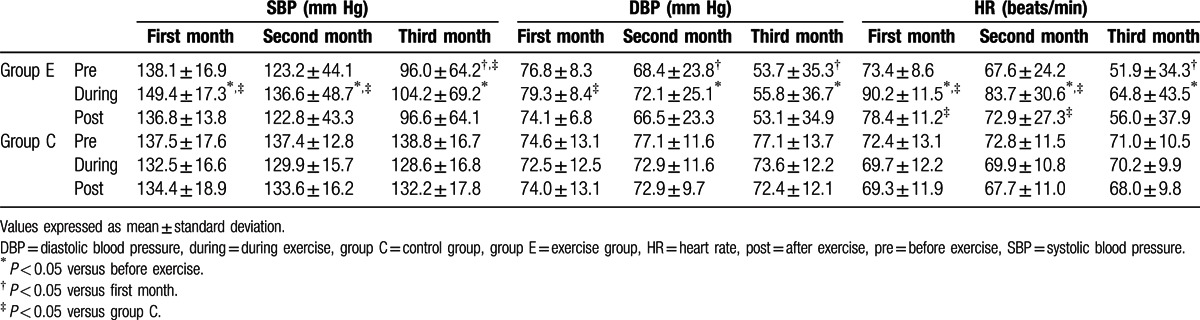
The changes of blood pressure and heart rate of hemodialysis patients who were prescribed a 3-month exercise program (group E) or not prescribed exercise (group C) and completed the study.

### 3.2 Biochemical and anthropometric parameters

Biochemical and anthropometric parameters before and after exercise are presented in Table 3. In group C, there was no obvious difference in biochemical data. The dialysis efficiency (Kt/V), normalized protein catabolic rate (nPCR), and inflammatory cytokine level were also not different after 3 months. In group E, although there were no obvious differences in the KT/V, nPCR, body weight, blood pressure, and biochemical data including iPTH, Ca, alanine aminotransferase, and hematocrit compared with baseline levels, there were statistically significant increases in serum albumin and BMI in patients after intradialytic aerobic cycling exercise. Furthermore, after intradialytic aerobic cycling exercise, the patients had lower levels of serum inflammatory cytokines, including hs-CRP and IL-6, after 3 months of exercise (Table 3).

**Table 3:**
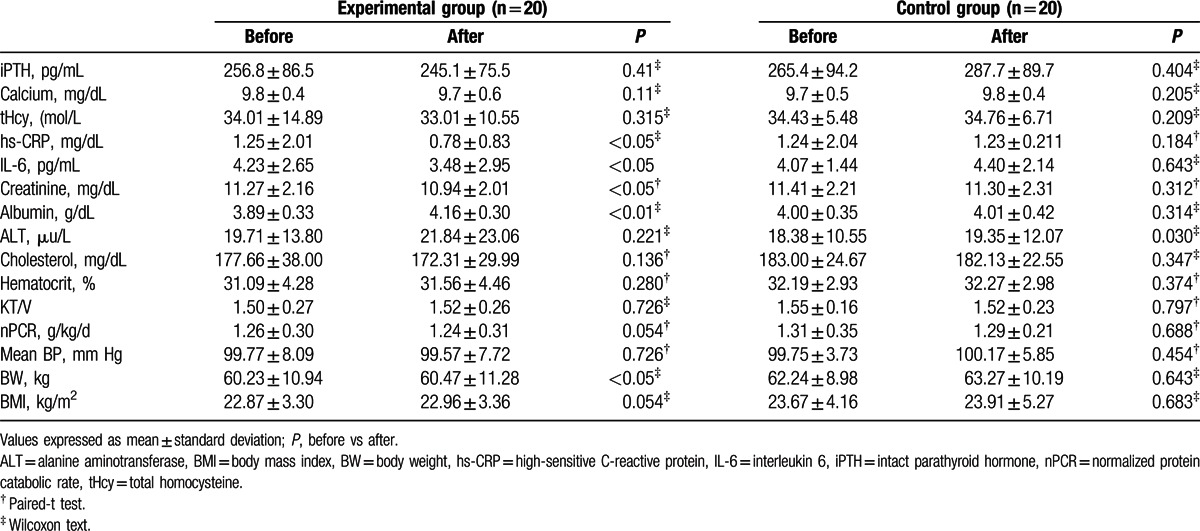
Comparison of clinical characteristics and biochemistry data of hemodialysis patients before and after 3 months of exercise.

### 3.3 Characterization of endothelial progenitor cells

**Figure 2:**
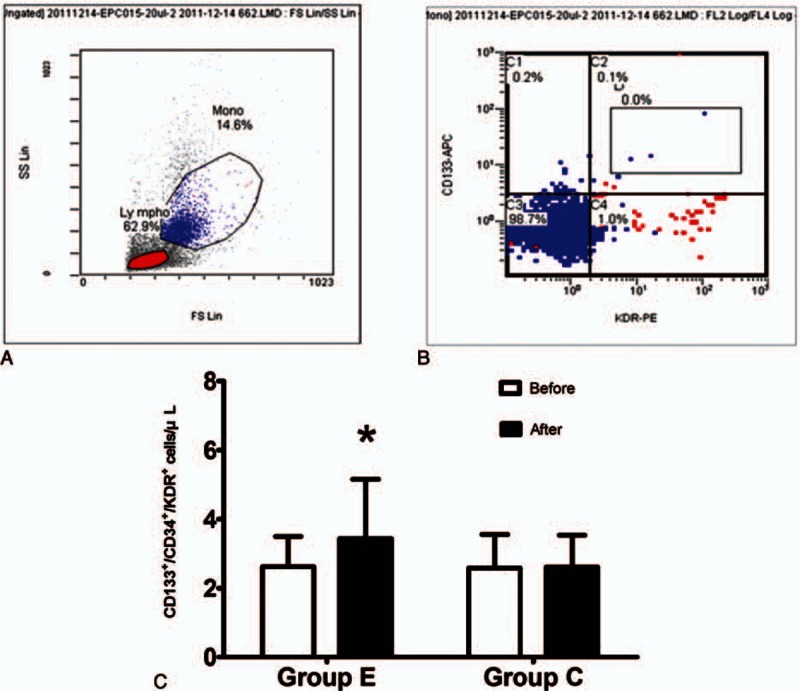
Characterization and changes of fluorescence-activated cell sorting (FACS)-sorted endothelial progenitor cells (EPCs) from a peripheral blood monocyte suspension. A, Cell viability gates were set as indicated on the peripheral blood monocyte suspension. Fractions enclosed by squares in (A) were CD133 and CD34-positive stem cell markers, and they were further characterized according to an endothelial cell-related marker, kinase insert domain-conjugating receptor (KDR). B, CD133, CD34, and KDR-positive EPCs were gated and sorted. C, Changes in the number of EPCs at the baseline and after 3 months in patients in the exercise group (group E) and control group (group C). ∗*P* < 0.05 according to a paired *t* test or Wilcoxon test.

At the end of the intradialytic aerobic cycling exercise study period, the patients in group E exhibited statistically significant increases in circulating CD133,CD34, and KDR-positive EPCs *(P* < 0.05) compared with the baseline measurements, whereas the number of circulating EPCs did not change in group C (Fig. 2).

### 3.4 Bone mineral density

**Figure 3:**
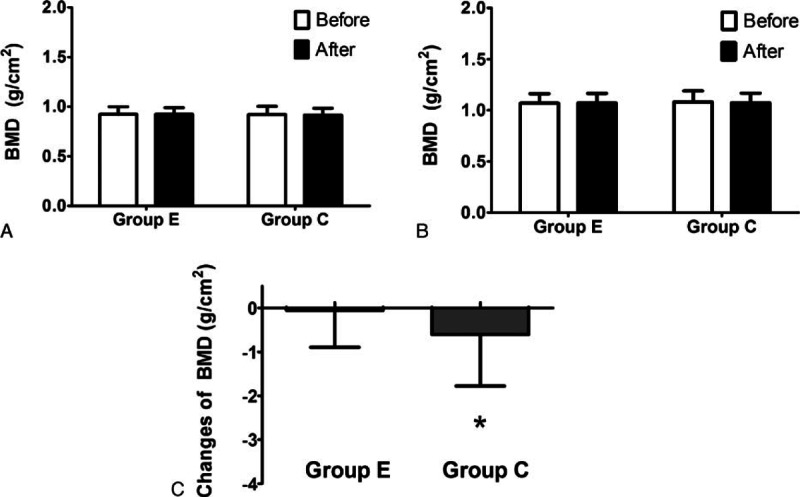
Changes in bone mineral density after 1 year of follow-up. There was no significant difference in the bone loss at the (A) femoral neck and (B) lumbar spine (L1–L4) between the 2 groups. C, Patients undergoing intradialytic exercise had negligible loss of bone mineral density at the femoral neck after 1 year of follow-up compared with the control patients (*P* < 0.05). ∗*P* < 0.05 according to a paired *t* test or Wilcoxon test.

One year after the initiation of the study, bone loss at the femoral neck was significantly attenuated in the patients who performed intradialytic aerobic cycling exercise compared with those who did not (*P* < 0.05; Fig. 3). However, there was no significant difference in bone loss in the lumbar spine (L1–L4) between the 2 groups.

### 3.5 The 6-minute walk test

**Figure 4:**
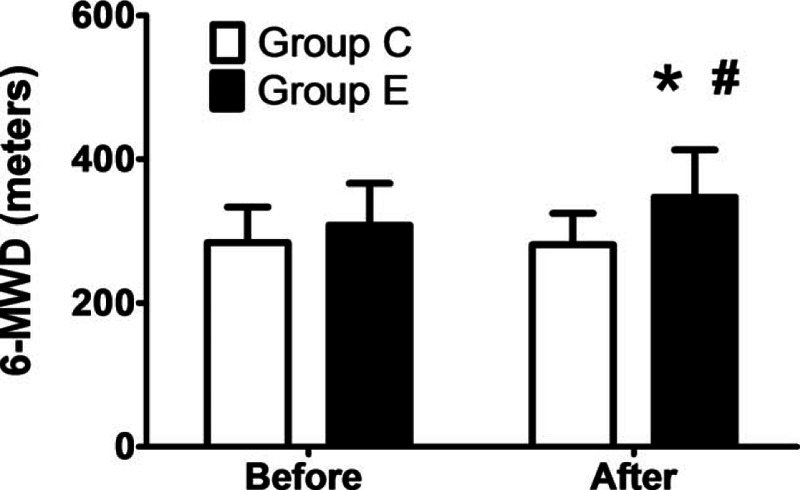
Changes in 6-minute walking distance (6-MWD) at the baseline and after 3 months. The patients in the exercise group (group E) had a significantly greater 6-MWD after the 3-month program compared with the baseline value (*P* < 0.05), but there was no significant change in the 6-MWD in the patients in the control group (group C). ∗*P* < 0.05 according to a paired *t* test or Wilcoxon test.

**Figure 5:**
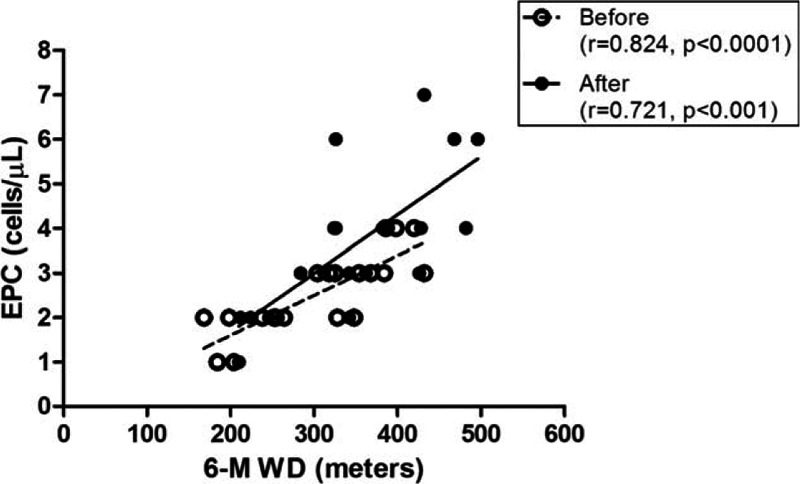
Correlation of endothelial progenitor cell (EPC) count with the 6-minute walking distance (6-MWD) at the baseline and after 3 months. The number of EPCs significantly correlated with the 6-MWD both at the baseline and after 3 months (*r* = 0.824 and 0.721, *P* < 0.0001 and 0.001, respectively, n = 20).

The patients in group E had a significantly greater 6-MWD after the intradialytic aerobic cycling exercise program compared with their baseline scores (*P* < 0.05), but there were no significant changes in the 6-MWD in group C (Fig. 4). Furthermore, the number of EPCs was significantly correlated with the 6-MWD both at the baseline and after 3 months (*r* = 0.824 and 0.721, *P* < 0.0001 and 0.001, respectively; Fig. 5) (n = 20).

## 4 Discussion

The present study demonstrated that the 3-month intradialytic aerobic cycling exercise program was effective in increasing serum albumin and circulating EPC numbers, reducing inflammatory markers (IL-6 and CRP), improving cardiovascular endurance and functional capacity (6-MWD), and attenuating the loss of femoral neck bone density in HD patients. There were no complications, and the exercise was tolerated well by all participants. Accordingly, intradialytic aerobic cycling exercise is a safe and economic approach to alleviating inflammation and improving nutrition, bone density, cardiovascular endurance, and functional capacity in HD patients.

Uremic patients often have chronic inflammation combined with malnutrition and atherosclerosis, a combination termed malnutrition, inflammation, atherosclerosis (MIA) syndrome.^[16]^ Although the underlying mechanisms of MIA syndrome are complex and not fully understood, it is known to be an important contributor to morbidity and mortality. In our study, both the exercise and control groups had high normal levels of IL-6 and hs-CRP at the baseline, indicating that ESRD patients are prone to inflammation. After 3 months of aerobic exercise, a significant decrease in the plasma levels of IL-6 and hs-CRP, and a simultaneous increase in plasma albumin levels suggested that the exercise program may attenuate chronic inflammation and therefore improve the nutritional status in HD patients.^[17,18]^ Exercise has anti-inflammatory effects on the immune system.^[19]^ It can induce anti-inflammatory cytokines and may selectively deplete circulating CD16+ monocytes through transient spikes in endogenous glucocorticoid.^[20]^ In addition, physical activity was reported to ameliorate Toll-like receptor (TLR)-dependent inflammation by reducing the cell surface expression of TLR4 and then blunting the lipopolysaccharide-induced inflammatory response.^[21]^ Furthermore, exercise may prevent the reactive oxygen species (ROS)-mediated breakdown of nitric oxide (NO), and increase the NO bioavailability to reduce the oxidative stress in chronic kidney disease (CKD) patients.^[22,23]^ All of these cellular mechanisms might contribute to the anti-inflammatory effect of exercise. Inflammation can induce signaling cascades that result in high protein catabolic rates, low protein synthesis, and, consequently, malnutrition. In group E, body weight had significantly increased after the 3-month intradialytic aerobic cycling exercise. Taken together, these findings support the notion that the anti-inflammatory effects of intradialytic aerobic cycling exercise may attenuate inflammation and protein-energy wasting in HD patients.^[24–26]^

High proinflammatory cytokine levels may contribute to bone loss and fracture in patients with CKD.^[27]^ Exercise may promote bone growth and suppress bone loss through direct mechanisms by increasing mechanical stress on bones. In addition, inflammatory cytokines are potent activators of receptor activator of nuclear factor kappa B ligand / receptor activator of nuclear factor kappa B (RANKL/RANK)-activated osteoclastogenesis.^[28]^ Exercise may inactivate the RANKL/RANK pathway by ameliorating inflammation and preventing bone loss in HD patients. In this study, the changes of blood pressure and heart rate during intradialytic aerobic cycling exercise had attenuated gradually and the pre-exercise blood pressure also had decreased after the 3-month training. The cardiovascular endurance and functional capacity had improved after the 3-month intradialytic aerobic cycling exercise. The patients in the exercise group also showed improvements not only in their nutritional condition but also in their BMD. Our data indicate that intradialytic aerobic cycling exercise has beneficial effects on HD patients’ BMD, cardiovascular endurance, and physical performance (6-MWD). Compared with the control group, the patients who underwent intradialytic aerobic cycling exercise had negligible loss of BMD in the femoral neck after a 1-year follow-up. However, this difference was not observed in the lumbar spine, probably because of the spine's position during intradialytic aerobic cycling exercise.^[29]^ Exercise can increase muscle mass. Lean body mass is independently associated with bone mass and BMD, regardless of age, sex, and postmenopausal women.^[30]^ Exercise can affect bone turnover from both directly mechanical force and indirectly through the activation of several endocrine axes. Several molecules secreted by adipose tissue (adipokine) and muscle (myokine) in response to exercise are involved in the fine regulation of bone turnover.^[31]^ Apart from PTH, vitamin D, cortisol, and exercise-induced myokine had profound effects in enhancing bone mass. In an in vitro study, it was shown that irisin can improve bone geometry even when given at a lower dose without browning response of adipose tissue.^[32]^ Obviously, the improved BMD in our patients with intradialytic exercise is not by altering the metabolism of calcium, phosphate, or PTH. The increase of femoral neck bone density may be related to the direct mechanical force, or the effects of adipokines or myokines during exercise.

Recently, cross-talk between skeletal muscles and host immunity has been proposed. Persistent inflammation and immune dysregulation in HD patients may result in loss of skeletal muscle mass, and also impairment of muscle strength and functional performance. Exercising mechanically stretched muscle fiber may alter the local inflammatory responses that affect structural and functional adaptation, remodeling, and repair processes in skeletal muscles.^[33]^ Bone strength is dependent on both through BMD, which is determined by peak bone mass, the amount of bone loss, and bone quality. Bone quality in this case refers to bone architecture, turnover, mineralization, accumulation of microdamage, and collagen properties.^[34–36]^ In CKD patients, high bone turnover was associated with greater severity of cortical porosity, and low turnover was associated with lower trabecular volume and thinner cortices.^[37]^ Thus, both high turnover and low turnover bone disorders entail low BMD.^[38]^ The attenuated bone loss observed after the exercise program in this study may have at least partially alleviated the fracture risk. With better nutrition and higher BMD, it is not surprising that our patients in the exercise group had higher functional capacity, as reflected by the more favorable cardiovascular endurance and 6-minute walk test results. The direct action of mechanical stress on bone and muscles exerted through exercise, together with the resulting anti-inflammatory effects and subsequent responses, may have contributed to the improvement in BMD and physical performance.

Endothelial progenitor cells, mobilized from the bone marrow, function as an endogenous agent for repairing the vascular endothelium, contribute to angiogenesis, and combat atherosclerosis.^[39,40]^ In CKD, the reduced number and impaired function of EPCs may play a critical role in contributing to the cardiovascular events in patients on maintenance HD.^[9,41,42]^ In CKD patients, the number and function of EPCs decline when renal function deteriorates and are correlated with the risk of cardiovascular disease.^[43,44]^ Uremic toxin greatly inhibits the differentiation and migration of EPCs. Hence, removing uremic toxin through dialysis may ameliorate the EPC level and improve endothelial function.^[45]^ Statins and angiotensin-converting enzyme inhibitors can increase the level and improve the function of circulating EPCs.^[46,47]^ In our study, the number of circulating EPCs was small at the baseline in both the exercise and control groups. The intradialytic aerobic cycling exercise in our study significantly increased the number of EPCs in the HD patients. This is consistent with previous studies that found that exercise, especially ischemic or subischemic exercise, increased the number of EPCs in healthy subjects and in patients with cardiovascular disease.^[11,48]^ It was proposed that this positive effect of ischemic exercise on EPC levels is due to a hypoxic status and increased shearing stress in the vasculature, which facilitate mobilizing pre-existing EPCs.^[49,50]^ In addition, inflammation of endothelial cells causes the dysfunction of EPCs.^[51]^ Hence, the anti-inflammatory effects of exercise may contribute to the increased EPC number and improvement of EPC function.^[52]^ We demonstrated a strong correlation between EPCs and the 6-MWD in HD patients. Thus, the intradialytic aerobic cycling exercise provided beneficial effects; however, whether these effects are mediated by EPCs and whether our program has therapeutic potential require further investigation.

Reducing inflammation, improving nutrition, and ameliorating CKD and MBD in HD patients are crucial objectives for nephrologists. We first demonstrated that intradialytic aerobic cycling exercise can improve BMD and showed correlations between EPCs and 6-MWDs. All participants in the current study were compliant, with none leaving early or experiencing adverse effects. The fact that intradialytic exercise involves simultaneous exercise and dialysis while under the supervision of doctors and machine surveillance makes it a feasible and applicable treatment option for HD patients. The value of intradialytic exercise has been investigated in various studies, with the majority demonstrating positive effects. Although many studies have demonstrated that intradialytic exercise can increase muscle blood flow and thereby increase solute removal during HD, no significant difference was found in our study. Adequate dialysis using a high-flux dialyzer combined with a relatively mild intensity and duration of exercise may be the main reason. Although the therapeutic effects of intradialytic aerobic cycling exercise have been demonstrated in this study, its application to all HD patients requires further research because of the relatively short observation time and small sample size. Nevertheless, intradialytic aerobic cycling exercise was demonstrated to be a feasible new adjuvant intervention for HD patients.

Some limitations of this study should be addressed. First, our sample was relatively small, which may have limited our ability to detect differences due to lower statistical power. Our study also lacked a sham exercise control activity because of the single geographic site used, and the unblinded assessment of physical performance measure (6-minute walk test). Second, the 3-month intervention may have been too short to improve the bone marrow density and other risk factors including markers of inflammation. Additional research with larger study population and longer interventions will be needed to more thoroughly assess the benefits associated with intradialytic exercise training.

In conclusion, intradialytic aerobic cycling exercise with moderate intensity is a safe, low-cost, and efficient approach to improving the nutritional status, cardiovascular endurance, and functional capacity of HD patients while reducing their cardiovascular risk. Along with these benefits, we found simultaneous attenuation of inflammatory responses, which might contribute to these beneficial effects of exercise. Large-scale randomized controlled trials are required to optimize the intradialytic exercise program for HD patients.
